# Adherence to Artemisinin Combination Therapy for the treatment of uncomplicated malaria in the Democratic Republic of the Congo

**DOI:** 10.12688/f1000research.6122.2

**Published:** 2015-04-08

**Authors:** M. Ruby Siddiqui, Andrew Willis, Karla Bil, Jatinder Singh, Eric Mukomena Sompwe, Cono Ariti

**Affiliations:** 1 MSF-UK, London, EC1N 8QX, UK; 2 MSF-Canada, Toronto, ON, M5S 2T9, Canada; 3 MSF-OCA, Amsterdam, P.O Box 10014, Netherlands; 4 MSF-OCA, Lubumbashi, Katanga, Congo, Democratic Republic; 5 Katanga Ministry of Health, Lubumbashi, Katanga, Congo, Democratic Republic; 6 London School of Hygiene and Tropical Medicine, London, WC1E 7HT, UK

**Keywords:** Adherence, artemisinin, compliance, treatment failure, artesunate-amodiaquine, side effects, malaria, Plasmodium falciparum

## Abstract

Between 2011 and 2013 the number of recorded malaria cases had more than doubled, and between 2009 and 2013 had increased almost 4-fold in MSF-OCA (Médecins sans Frontières – Operational Centre Amsterdam) programmes in the Democratic Republic of the Congo (DRC). The reasons for this rise are unclear. Incorrect intake of Artemisinin Combination Therapy (ACT) could result in failure to treat the infection and potential recurrence. An adherence study was carried out to assess whether patients were completing the full course of ACT.

One hundred and eight malaria patients in Shamwana, Katanga province, DRC were visited in their households the day after ACT was supposed to be completed. They were asked a series of questions about ACT administration and the blister pack was observed (if available).

Sixty seven (62.0%) patients were considered probably adherent. This did not take into account the patients that vomited or spat their pills or took them at the incorrect time of day, in which case adherence dropped to 46 (42.6%). The most common reason that patients gave for incomplete/incorrect intake was that they were vomiting or felt unwell (10 patients (24.4%), although the reasons were not recorded for 22 (53.7%) patients). This indicates that there may be poor understanding of the importance of completing the treatment or that the side effects of ACT were significant enough to over-ride the pharmacy instructions.

Adherence to ACT was poor in this setting. Health education messages emphasising the need to complete ACT even if patients vomit doses, feel unwell or their health conditions improve should be promoted.

## Introduction

Despite a wealth of natural resources, the Democratic Republic of the Congo (DRC) has one of the lowest per capita incomes in the world ^1^ and in 2014 ranked 186th (out of 187 countries) in the United Nations Development Programme’s human development index ^2^.

Médecins Sans Frontières-Operational Centre Amsterdam (MSF-OCA) has been working in the provinces of North Kivu and South Kivu since the early 1990s and in Katanga since 2003. MSF-OCA operates health programmes in Mweso and Walikale in North Kivu; Baraka and Kimbi Lulimba in South Kivu; and Shamwana, Katanga (DRC).

**Figure 1.**
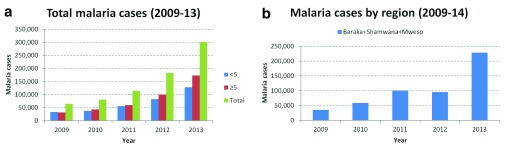
Confirmed malaria cases in MSF-OCA programmes and in fixed programmes in Baraka, Shamwana and Mweso in 2009–2011.

Between 2011 and 2013, the number of recorded malaria cases more than doubled and between 2009 and 2013, increased almost 4-fold in MSF-OCA programmes in DRC ( Figure 1a). The number of projects that MSF-OCA supported between 2009 and 2013 has however varied during that time. Hence, restricting the analysis to the fixed programmes only (in Baraka (South Kivu), Shamwana (Katanga) and Mweso (North Kivu)), the general trend still appears to be a marked increase in malaria cases ( Figure 1b).

**Figure 2.**
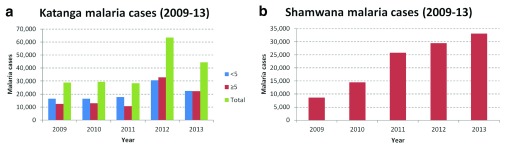
Confirmed malaria cases in Katanga and in Shamwana, Katanga in 2009–2011.

In Katanga the general trend is less clear for malaria cases. However this is likely due to the variable numbers of projects supported between 2009 and 2013 ( Figure 2a). Focussing on the fixed project in Shamwana, there has been a marked increase in malaria cases ( Figure 2b).

In 2013 MSF clinic staff in Shamwana began to identify patients returning to the clinic with complicated severe malaria after having already been prescribed the anti-malarial treatment. It was not known if these were treatment failures or re-infections.

This was an alarming increase in malaria incidence which cannot be explained by displacement of non-immune populations from non-endemic regions (mountains) to endemic malaria regions as the populations have been relatively stable since 2008.

The World Health Organisation (WHO)-recommended strategies for malaria control fall into two major areas: prevention and case management ^3^. These strategies work against both the transmission of the parasite from mosquito vector to humans (and from humans to mosquitoes) and the development of illness and severe disease in humans ^3^. The former is achieved in Shamwana through the distribution of long-lasting insecticide-treated bednet (LLIN) in a targeted manner (pregnant women and under 5 year old malaria patients) and the latter through Artemisinin Combination Therapy (ACT) of confirmed malaria cases (the national protocol of DRC is Artesunate and Amodiaquine (ASAQ) administered in a fixed dose combination (FDC) as a first-line ACT). These strategies do not seem to be having an impact on malaria incidence in MSF settings in DRC.

The current study explored whether this increase in cases could be explained by patients failing to complete the full three day course and adhering to the time schedule and dosage prescribed for ASAQ. Poor adherence increases drug pressure and thus the risk of developing resistance. Moreover, incorrect or incomplete intake could result in failure to treat the infection and potential recurrence.

## Materials and methods

### Study design

The study was conducted in Shamwana, Katanga, DRC. Katanga has an average temperature of 24°C and two seasons, rainy between November and March and dry from April to October. MSF-OCA has been working in the province of Katanga since 2003. MSF-OCA supports Shamwana hospital to provide free secondary health care and supports more than four health centres. The MSF catchment population included Shamwana town and the nearby village of Nsangwa.

### Sample size

For previous ACT adherence assessments, adherence was always approximately 60% ^4– 9^. The sample size was therefore based on the conservative estimated adherence of 50% (in order to obtain the maximum sample size). With a precision of 10% and an α-error of 5%, n=97 patients were required. Assuming a proportion of patients lost to follow-up or withdrawn of 20%, the total sample size needed for the home visits was thus n=117 patients. Similarly, a sample size of 117 was calculated for the exit interviews.

To prevent bias due to a change in prescribing and educating behaviour by health staff, the health staff were informed that a study would start but the exact purpose of the study (measuring patient adherence) was at no time revealed to them. The explanation was that this was a mapping exercise of where patients attending the health centre were mainly coming from.

## Study procedure and questionnaires

### Centre questionnaire

At the health centre level, all patients were interviewed using a short “centre”-questionnaire. This included the patient’s name, age, sex, the number and type of prescriptions, and the patient's address in sufficient detail.

### Home questionnaire

Study patients for the “home”-questionnaire were selected through systematic sampling of eligible patients (aged more than 1 year with a positive malaria rapid diagnostic test (RDT) result that had been prescribed ASAQ) from the “centre”-questionnaires. Study patients were visited at home on Day 3 (the health centre visit was on Day 0) and interviewed using the “home” questionnaire to assess patient adherence. Socio-demographic questions about the household were followed by a systematic account of how the pills were taken. A correct treatment schedule instrument was used to guide the questions on adherence. Lastly, some questions about knowledge of malaria cause and prevention were asked. Any sick patients were referred to the health centre.

### Exit questionnaire

After all ‘home’-questionnaires were completed, exit interviews were carried out at the health centre using the “exit”-questionnaire to provide further information on patient/caretaker understanding of ACT and pharmacy dispensing practices.

## Adherence definitions

A dose was considered correctly taken if according to the patient or caretaker’s verbal account the prescribed amount of pills was taken and it was neither spat out nor vomited.

There are no standard definitions for adherence so the following were used ( Table 1).

**Table 1.** Final classification scheme of patients, according to the treatment intake and the presence of the medication blister packaging.

Intake	Blister packaging	Final classification
**Incomplete**	Present	Certain non-adherence (pills remaining)
**Incomplete**	Not present	Probable non-adherence (patient describes incomplete number of pills taken)
**Incorrect**	Present or not	Probable non-adherence (patient describes incorrect time schedule or dosage)
**Correct**	Present or not	Probable adherence (patient describes correct number of pills taken, time schedule and dosage)

**Certain incomplete intake or certain non-adherence**: Any patient that showed blister packaging still containing any ASAQ pills was certain to have taken an incomplete treatment. There was absolute certainty that the treatment schedule was not completed.

**Probable incomplete intake or probable non-adherence**: According to the patient/caretaker’s account, all pills were not taken so this patient was probably non-adherent. The blister packaging presented was empty or could not be shown.

**Probable incorrect intake or probable non-adherence**: If according to the patient/caretaker’s account ASAQ was not taken following the prescribed time schedule or dosage, it was assumed the patient took the complete treatment but in an incorrect way. The blister packaging presented was empty or could not be shown.

**Probable correct intake or probable adherence**: Only those patients who stated they had taken the complete treatment schedule, following the treatment protocol exactly were considered “probably adherent” (this was not ‘certain’ as each dose had not been observed). The blister packaging presented was empty or could not be shown.

## Data management and analysis

Data for each subject were entered into EpiData 3.1 software ^10^. Data were summarised by number and percentages for categorical variables. Continuous variables were summarised using means, medians and ranges. Adherence status was calculated and expressed as a percentage. Confidence intervals were calculated using binomial and multinomial formulae. To calculate the associations between risk factors and adherence status logistic regressions was used and the results expressed as odds ratios and 95% confidence intervals. Data analysis was conducted using Stata 13.0 ^11^.

## Results

### Survey profile

In total, 274 people completed the ‘centre’ questionnaires. Among those, 150 (54.7%) were diagnosed with malaria and given a prescription for ACT. Of these malaria patients 17 were aged <1 year so were excluded. Onehundred and seventeen (42.7%) of the 133 (48.5%) patients that satisfied the inclusion criteria were selected for home visits as these were the maximum that could be achieved during daily visits. However, only 108 (39.4%) of these eligible patients completed home questionnaires. The remaining 9 (3.3%) patients were considered lost to follow-up ( Table 2). A total of 117 exit questionnaires were completed for patients who had received an ACT prescription in one day ( Table 2). Nobody refused to participate in the study.

**Table 2.** Patient flow through study.

Patient flow through study	Number of patients (N)	Proportion of centre questionnaires (%)
**Centre questionnaires**	274	100.0
**RDT-positive and ACT prescription**	150	54.7
**ACT prescription and eligible**	117	42.7
Lost to follow up*	9	3.3
**Home questionnaires**	108	39.4
**Exit questionnaires**	117	

*Reasons for loss to follow up included; temporarily out of the village (6), wrong address (1), age was <1 year (1), household already visited. No patients were lost to follow-up due to admission to hospital.

### Socio-demographic description

The mean age of the patients was 10.5 years and ranged from 1 to 57 years (median 5 years, Q25–Q75: 2–12.5, Table 3). Sixty (55.6%) patients were aged ≤5 years. The sex ratio was 0.7 (male/female 45/61). The age of the 28 patients that responded to the questionnaire ranged from 9 to 57 years (though parents/caretakers were present to give informed consent for the eight respondents that were aged <18 years). The age of the patients for whom a parent/caretaker responded ranged from 1 to 17 years. The most common caretakers were parents (74, 92.5%).

**Table 3.** Socio-demographic description of the study population (patients and caretakers).

Socio-demographic factor	N	%		Socio-demographic factor	N	%
**Age groups (years)**				**Origin**		
≤1 (infant)	13	12.0%		Resident	67	62.0%
2–5 (young child)	47	43.5%		IDP	41	38.0%
6–13 (adolescent)	23	21.3%		**Total**	**108**	**100.0%**
≥14 (adult)	25	23.2%				
**Total**	**108**	**100.0%**		**Sex**		
				Male	45	41.7%
**Mean age (years)**	10.5			Female	61	56.5%
**Median age (years)**	5			Not known	2	1.8%
**Range (years)**	1-57			**Total**	**108**	**100.0%**
						
**Caretaker relation to patient**				**Highest education level of patient**		
Patient	28	25.9%		Can’t read/write	46	42.6%
Father/Mother	74	68.5%		Primary incomplete	47	43.5%
Grandfather/Grandmother	5	4.6%		Primary complete	4	3.7%
Brother/Sister	1	1.0%		Secondary incomplete	11	10.2%
**Total**	**108**	**100.0%**		Secondary complete	0	0%
				Higher incomplete	0	0%
				Higher complete	0	0%
				**Total**	**108**	**100.0%**

Education levels of the patients were low with no patients completing secondary education or higher ( Table 3). The education levels of the patient/caretakers that attended the clinic (i.e. those likely administering the ACT treatment to children) were not assessed.

The mean household size was 8.6 members and ranged between 1 and 26 (median: 8, Q25–Q75: 5–10.5). The mean number of children aged <5 years per household were 2.6 and ranged between 0 and 13 (median: 2, Q25–Q75: 2–3) ( Table 4). The main profession of heads of households was subsistence farming alone (46, 42.6%), and this occupation was named in combination with others for an additional 40 households (86, 79.6%).

**Table 4.** Socio-demographic description of the study households.

Demographic data	Households	
**Number of household members**	**N**	**%**
1–4 members	29	26.9%
5–8 members	32	29.6%
9–12 members	34	31.5%
>13 members	13	12.0%
**Total**	**108**	**100.0%**
		
**Mean household size (members)**	**8.6**	
**Median household size (members)**	**8**	
		
**Number of children <5 in households**	**N**	%
0 children	9	8.3%
1 children	16	14.8%
2 children	34	31.5%
3 children	29	26.9%
4 children	10	9.3%
5 children	3	2.8%
6 children	5	4.6%
≥10 children	2	1.8
**Total**	**97**	**100.0**
		
**Mean number of children <5 per household**	**2.6**	
**Median number of children <5 per household**	**2**	
		
**Profession of head of household**	**N**	%
Subsistence farmer	46	42.6%
Subsistence farmer + Daily worker	8	7.4%
Subsistence farmer + Other profession *	6	5.6%
Subsistence farmer + Farmer for trading	5	4.6%
Health worker	5	4.6%
No work	5	4.6%
Workman	4	3.7%
Subsistence farmer + Hunter	4	3.7%
Subsistence farmer + Teacher	3	2.8%
Subsistence farmers + Other professions †	14	13.0%
Other professions ‡	7	6.5%
Missing	1	0.9%
**Total**	**108**	**100.0%**

*Includes blacksmith (1), coalman (1), dressmaker (1), mason (1), sells fish oils (1), sells wood (1). †Includes health worker (2), student (2), no work (2), workman (2), trader (2), daily worker + other (1), daily worker + teacher (1), trader + other (1), farmer for trading + workman (1). ‡Includes guard (2), no work + other (1), daily worker + health worker (1), trader (1), farmer for trading + daily worker (1), farmer for trading + trader (1).

### Patient adherence

A total of six (5.6%) patients had one or more pills left at the time of the home visit on Day 3 when all pills should have been taken ( Table 5). They were defined as certainly non-adherent. Thirtyfive (32.4%) patients were defined as probably non-adherent after describing an incomplete intake (including eight patients that took ASAQ pills on Day 3) and 67 (62.0%) patients were described as probably adherent after describing a complete and correct intake ( Table 5). Merging the two non-adherent groups gave 41 (38.0%) non-adherent patients.

**Table 5.** Adherence to currently used ACT treatment (ASAQ) for uncomplicated malaria.

Calculation of adherence	Incomplete/incorrect intake described	Complete/correct intake described	Total
No blister	21	34	**55**
Blister empty	14	33	**47**
Blister with pills	6	0	**6**
**Total**	**41**	**67**	**108**
			
**Classification of adherence**	**Number of patients (N)**	**Proportion (%)**	**95% CI**
Certain non-adherence	6	5.6%	1.24-9.88%
Probable non-adherence	35	32.4%	23.58-41.23%
Probable adherence	67	62.0%	52.88-71.99%
**Total**	**108**	**100.0%**	
			
**Adherence status**	**Number of patients (N)**	**Proportion (%)**	**95% CI**
Non-adherent	41	38.0%	28.81-47.12%
Adherent	67	62.0%	52.88-71.99%
**Total**	**108**	**100.0%**	

However it should be noted that in order to analyse these data, assumptions had to be made for missing data, primarily an assumption had to be made for 25 patients on Day 0 that ASAQ had been taken only once that day (at the clinic) and had been taken at the same time as the dose on Day 1 and Day 2 (which actually matched each other in all cases).

This adherence was calculated based on the number of pills taken, the number of times pills were taken per day and the number of days completed. However the timing of the daily dose and whether the medication was retained (not vomited or spat out) are also important. Both could result in a reduction of anti-malarial activity during the treatment phase and potential treatment failure. Using this stricter definition of adherence, 21 patients that had been defined as probably adherent became probably non-adherent (data not shown). Twelve (57.1%) of these patients did not take doses at the correct time of day, eight (38.1%) patients vomited or spat their pills and one (4.8%) patient vomited or spat their pills and also did not take doses at the correct time of day.

### Reasons for incomplete, incorrect or correct intake

Reasons for 1) pills remaining or 2) a description of incorrect intake or 3) a description of correct intake were noted for each patient. However, 22 (62.9%) of the 35 probably non-adherent patients had missing data for reasons for incorrect intake and 19 of the 67 (28.4%) probably adherent patients had missing data for reasons for correct intake.

This analysis only considered the individuals classified into the appropriate categories (non-strict adherence). The main reason for pills remaining (incomplete treatment intake) were forgetting to give/take the treatment and patient didn’t feel better/treatment wasn’t working + felt unwell (2, 33.3% respectively) ( Table 6). Unfortunately the design of the questionnaire did not allow analysis of whether a replacement dose was taken.

**Table 6.** Reasons given by patients for incomplete, incorrect and correct ACT intake.

Reason for ACT intake	Number of patients (N)	Proportion (%)
**Reasons given for incomplete intake (pills remaining)**		
Forgot to give/take	2	33.3
Patient didn’t feel better/Treatment wasn’t working + Felt unwell	2	33.3
Felt unwell	1	16.7
Patient didn’t feel better/Treatment wasn’t working	1	16.7
**Total**	**6**	**100.0**
		
**Reasons given for incorrect intake**		
Forgot to give/take	3	8.5%
Claims wrong instructions/dosage were given in the clinic	3	8.5%
Patient was vomiting	3	8.5%
Felt sick/unwell straight after taking the pills	1	2.9%
Thought patient would cure faster	1	2.9%
Thought patient would cure faster + Felt sick/unwell straight after taking the pills	1	2.9%
Thought patient would cure faster + Claims that wrong instructions/ dosage were given in the clinic + Felt sick/unwell straight after taking the pills	1	2.9%
Missing data	22	62.9%
**Total**	**35**	**100.0**
		
**Reasons given for correct intake**		
Correct instructions given at the clinic	32	47.8%
Given the same medicine before and knows how to take it + Correct instructions given at the clinic	13	19.4
Wanted to heal	1	1.5%
Child strong enough to take the medicine	1	1.5%
Given the same medicine before and knows how to take it + Was helped by the community health volunteer (ASC)	1	1.5%
Missing data	19	28.3%
**Total**	**67**	**100.0**

The most common reasons for incorrect intake were that the patient forgot to take/caretaker forgot to give the pills, claimed the wrong instructions were given at the clinic or the patient was vomiting (3, 8.5% respectively) ( Table 6).

Taken together the most common reason patients gave for incomplete and/or incorrect intake was that they were vomiting or felt unwell (10 patients (24.4%)).

The most common reasons for correct ACT intake was that the correct instructions were given at the clinic (32, 47.8%) and the patient/caretaker had been given the same medicine before and knew how to take it + Correct instructions were given at the clinic(13, 19.4%) ( Table 6).

### Assessment of possible risk factors

Univariate analyses of potential risk factors for non-adherence were explored (using the non-strict adherence classification). These included sex, weight classes (instead of age groups), education level of the patient, knowledge that mosquito bites can cause malaria, knowledge that LLINs prevent malaria and presence of observed LLINs in the household. Significant association with adherence was observed only for sex (χ ^2^ = 8.1, P= 0.017, Table 7). Women were more likely to adhere to ACT treatment. Sex remained significantly associated when considering strict adherence (χ ^2^ = 8.9, P= 0.031, Table 8) and when merging certain and probable non-adherence into a single ‘non-adherence’ category (χ ^2^ = 8.1, P= 0.004 for non-strict adherence only, Table 9, Table 10).

**Table 7.** Univariate analysis of sex and non-strict adherence (all categories), Pearson χ ^2^ = 8.10, P = 0.017 (Fishers exact P = 0.013).

	Sex		Total
**Non-strict adherence**	**Male**	**Female**	
Certain non-adherence	3 (6.6%)	2 (3.2%)	5 (4.7%)
Probable non-adherence	21 (46.7%)	14 (23.0%)	35 (33.0%)
Probable adherence	21 (46.7%)	45 (73.8%)	66 (62.3%)
**Total**	**45 (100.0%)**	**61 (100.0%)**	**106 (100.0%)**

**Table 8.** Univariate analysis of sex and strict adherence (all categories), Pearson χ ^2^ = 8.91, P = 0.031 (Fishers exact P = 0.026).

	Sex		Total
**Strict adherence**	**Male**	**Female**	
Certain non-adherence	3 (6.6%)	2 (3.3%)	5 (4.7%)
Probable non-adherence (incomplete intake)	21 (46.7%)	14 (23.0%)	35 (33.0%)
Probable non-adherence (incorrect intake)	5 (11.1%)	16 (26.2%)	21 (19.8%)
Probable adherence	16 (35.6%)	29 (47.5%)	45 (42.5%)
**Total**	**45 (100.0%)**	**61 (100.0%)**	**106 (100.0%)**

**Table 9.** Univariate analysis of sex and non-strict adherence (all categories), Pearson χ ^2^ = 8.10, P = 0.004 (Fishers exact P = 0.008).

	Sex		Total
**Non-strict adherence**	**Male**	**Female**	
Non-adherence	24 (53.3%)	16 (26.2%)	40 (37.7%)
Adherence	21 (46.7%)	45 (73.8%)	66 (62.3%)
**Total**	**45 (100.0%)**	**61 (100.0%)**	**106 (100.0%)**

**Table 10.** Univariate analysis of sex and strict adherence (all categories), Pearson χ ^2^ = 1.5, P = 0.217 (Fishers exact P = 0.239).

	Sex		Total
**Strict adherence**	**Male**	**Female**	
Non-adherence	29 (64.4%)	32 (52.5%)	61 (57.5%)
Adherence	16 (35.6%)	29 (47.5%)	45 (42.5%)
**Total**	**45 (100.0%)**	**61 (100.0%)**	**106 (100.0%)**

These risk factors were analysed in a logistic regression where the sex of the patient only was significantly associated with adherence ( Table 11).

**Table 11.** Multivariate regresssion of potential risk factors to ACT non-adherence.

Risk factors	Non-adherent (n=40)	%	OR	95% CI	P value
**Patient sex**					
**Male (n=45)**	24	53.3%			
**Female (n=61)**	16	26.2%	2.86	1.21-6.76	**0.017**
**Patient weight**					
**Infant (n=7)**	2	28.6%			
**Young child (n=65)**	26	40.0%	1.16	0.19-7.23	0.875
**Adolescent (n=17)**	7	41.2%	0.82	0.11-6.12	0.847
**Adult (n=19)**	5	26.3%	1.88	0.28-13.88	0.537
				
**Education level**					
**Illiterate (n=46)**	21	45.7%			
**Any education (n=62)**	20	32.3%	1.80	0.74-4.39	0.197
					
**Malaria caused by mosquito bites**					
**Not mentioned (n=42)**	20	47.6%			
**Mentioned (n=66)**	21	31.8%	1.98	0.64-6.19	0.238
					
**Bednet prevents malaria**					
**Not mentioned (n=36)**	15	41.6%			
**Mentioned (n=72)**	26	36.1%	0.85	0.25-2.92	0.795
					
**Bednet observed**					
**No (n=47)**	20	42.6%			
**Yes (n=61)**	21	34.4%	1.69	0.70-4.09	0.248

## Exit-questionnaires

### Socio-demographic description of the exit group

Patients or caretakers/parents in the exit population had broadly the same socio-demographic and clinical characteristics as those who were interviewed at home for the adherence study (i.e. sex of patient, caretaker relation to patient, education level of the patient).

The weight categories showed significantly higher infants and adults in the exit group (χ ^2^=9.34, P=0.026 for age group and χ ^2^=9.16, P=0.05 for weight class).

### Comprehension of ACT treatment schedule by exit group

In the exit-questionnaires group, a high number of the malaria patients or their respective caretakers (114, 97.4%) knew the name of the disease or could name the correct signs/symptoms of malaria ( Table 12). Similarly a high number (101, 86.3%) were able to identify the ACT pills among all the given pills and to indicate to the interviewer that they were an anti-malarial. Similarly, a high proportion of patients/caretakers (116, 99.2%) correctly said they would take the next dose the following day (though only six, 5.1% mentioned the time), correctly said they would take the next dose the day after (116 (99.2%), though only five, 4.3% mentioned the time), claimed that they would take all ACT pills and would therefore not have a balance at the end of the ACT course (117, 100.0%), claimed they would continue taking ACT treatment the following day even if their condition improved (115, 98.3%) and stated they would return to the clinic if their condition did not improve after 3 days (114, 97.4%) ( Table 12). This indicated a high level of understanding of ACT administration. However, only 87 (74.4%) patients/caretakers could repeat the number of times per day pills should be taken and 76 (65.0%) could repeat the number of pills to take ( Table 12).

**Table 12.** ACT treatment education and understanding in the exit group.

ACT treatment education and understanding	No. patients/ caretakers (N)	Proportion patients/ caretakers (%)
**Patient/caretaker understanding**	N=117	
**Understanding of malaria**		
**Able to name malaria**	60	51.3%
**Able to describe symptoms only**	54	46.2%
**Unable to describe disease or symptoms**	3	2.5%
**Recognition of ACT**		
**Can identify ACT**	101	86.3%
**Can identify ACT with other drugs**	10	8.6%
**Identifies other drugs only**	4	3.4%
**Doesn’t know**	2	1.7%
**Taken ACT previously**		
**0 times**	21	18.0%
**1-2 times**	31	26.5%
**3-4 times**	33	28.2%
**>5 times**	31	26.5%
**Missing data**	1	0.8%
**Understanding of ACT schedule**		
**Repeated instructions correctly-Days**	116	99.2%
**Repeated instructions correctly-Times**	87	74.4%
**Repeated instructions correctly-Pills**	76	65.0%
**1 ^st^ ACT dose observed in pharmacy**	101	86.3%
**Continuation of ACT treatment on Day 1 if condition improves**	
**No**	2	1.7%
**Yes**	115	98.3%
**Action if no improvement after 3 days**		
**Return to hospital**	114	97.4%
**Other**	1	0.9%
**Missing data**	2	1.7%
**Health centre role**		
**Patient was asked if s/he understood**	107	91.5%
**Patient was asked to repeat instructions**	41	35.0%
**Patient was given additional information (multiple responses possible):**		
**To return to clinic if pill vomited/spat**	59	50.4%
**May cause fatigue but must continue**	19	16.2%
**Complete the course**	2	1.7%
**Take at the same time each day**	2	1.7%
**Sleep under a bednet**	2	1.7%
**Eat**	1	0.9%
**No additional instructions**	32	27.4%

However, the Outpatient Department (OPD)/pharmacy performance was not consistent. Despite 107 (91.5%) of the patients/caretakers being asked if they had understood the instructions given to them, only 41 (35.0%) had been asked to repeat these instructions and only 90 (76.9%) were given additional information, mainly advised to return to the clinic to get a dose if they vomited or spat up a dose (51, 43.6%) and informed that ACT can cause fatigue but they must continue the treatment (16, 13.7%). Also a high but not optimal number of patients (101, 86.3%) had taken a dose of ACT at the clinic under supervision.

## Discussion

This study found that six (5.6%) patients were certainly non-adherent; therefore approximately one out of 18 patients did not complete the course of ACT treatment within the timeframe of 3 days. From the verbal account of the patients or caretakers, another 35 (32.4%) patients took the treatment in a different way to how it was prescribed (probable non-adherence). Therefore, only 67 (62.0%) patients could be considered probably adherent.

This did not take into account the patients that vomited or spat their pills or took the pills at the incorrect time of day. In that case the adherence dropped to 46 (42.6%). The effect of a decrease in anti-malarial activity that would follow vomiting/spitting or failure to take a timely dose is not clear but it is thought that this risks a period of monotherapy to the partner drug in the ACT combination. Advice about timing of ACT dosages is not clear but it is thought that a 3-hour margin either side of the expected timing is acceptable ^12^.

The most common reason patients mentioned for incomplete (67%) and incorrect (20%) intake was that they were vomiting or felt unwell. This appears to indicate that there was a poor understanding of the seriousness of the disease and the importance of finishing the treatment or that the side effects of ASAQ treatment were significant enough to over-ride the pharmacy instructions, unlike previous MSF studies using Coartem® ^8^.

More than a third of the study population claimed not to know the cause of malaria nor how to prevent malaria during home visits, but this was not significantly associated with non-adherence to ACT. Only sex of the patient was associated with adherence (males were significantly less likely to adhere to ASAQ). As the side effects were unlikely to affect males more than females, this may demonstrate an indifference to malaria in the male population, a common disease seen regularly in Shamwana. Similarly in Ethiopia, a study of factors associated with non-use of bed nets found that malaria was not perceived by the population to be a problem despite high prevalence of the disease ^13^.

When assessing the main reason for correct ACT intake, 45 (66.2%) patients claimed they were given the correct instructions at the OPD/pharmacy. When assessing the exit-questionnaires the quality of ACT prescriptions and the related instructions given at the OPD/pharmacy were less clear: 86.3% of the patients in the exit-questionnaires group were able to identify ACT as the anti-malarial treatment given to them amongst other concomitant treatments and 99.2% could correctly repeat the number of days that ACT should be taken. However, only 74.4% could repeat the number of times per day pills should be taken and 65.0% could repeat the number of pills to take.

Unfortunately the education level of the patients only was assessed, and not that of the caretakers. Therefore, any link between education level and adherence to antimalarials, which have been shown previously to be associated, could not be deduced ^5, 7^. Knowledge that mosquito bites cause malaria did not show a statistically significant association with ACT adherence unlike findings of an ACT adherence study in Bo, Sierra Leone ^8^. No association was seen between adherence and age despite some observations in MSF projects that adherence is poor amongst adolescents.

Three ACT adherence studies carried out in MSF malaria projects had similar findings; certain non-adherence was 22.9% and probable non-adherence 28.8% in a rural area of Sierra Leone, 18% and 29% in a remote area in South Sudan and 21% and 39% respectively in a refugee settlement in Zambia respectively ^6– 8^. These study settings had similar characteristics to Shamwana, such as a remote rural study area, low education level, treatment intake over 3 days, and treatment already implemented for a certain amount of time. Furthermore, our results are in line with a study carried out in two districts in Western Kenya that found a certain non-adherence of 31.7% and probable non-adherence of 4.2% ^14^ and another study in rural Sri Lanka using post-treatment interviews where 26% of patients self-reported defaulting. The main reasons given for not taking the entire regimen were side effects and disappearance of symptoms ^15^.

However, studies in Uganda and Ghana showed certain non-adherence to be as low as 7% and probable non-adherence to be 3% ^7, 16^. In Tanzania, non-adherence was as low as 25% ^17^. These higher adherences might be explained by the fact that adherence assessments were carried out after the successful introduction of a new malaria treatment. The introduction of a new, efficient treatment is always accompanied by special training for health workers, which is linked with greater motivation, and giving a more detailed verbal explanation to patients.

A recent systematic review found 37 studies that measured ACT adherence ^18^. However all had varying definitions of adherence and used different study designs and methods to measure adherence. Standardised methodologies for both self-report and bioassay measurements would improve the evidence base on ACT adherence and effectiveness.

Two different methods were used to measure adherence in patients: the pill count (observation of the blister pack) and a systematic questionnaire. Both assessment methods have their limitations: the pill count is a more objective measurement but gives incomplete information; the patient’s account is subjective and not verifiable but provides more information. By classifying patients as either certainly or probably non-adherent, the study accounted for the limitations of both methodologies.

This study has shown that ACT adherence in this setting is inadequate and may have contributed to the increase in malaria cases (if recrudescence was a major cause). It should be noted however that there is no recommended target for adherence levels. Artemisinin is the best drug available to treat malaria and currently there are no real alternatives. Artemisinin resistance has likely arisen in Cambodia due to the use of artemisinin monotherapy ^19^. Combination treatment with ACT when taken correctly reduces, but does not completely eliminate, the chance of resistant strains developing. Poor adherence could potentially expose greater numbers of parasites to the more slowly-eliminated partner drug in ACTs, increasing the risk of resistance to the partner drug ^20^. Once resistance has developed to the partner drug the ACT treatment would effectively become artemisinin monotherapy and hence render artemisinin vulnerable to resistance.

The effectiveness of ACT relies on both the efficacy of the drug components and on correct compliance. Adherence to ACT should not be taken for granted. At the community level, health communication campaigns that improve malaria knowledge and help them understand the importance of prevention and correct treatment should be carried out. At the clinic level, first ACT dose should always be observed at the pharmacy/OPD clinic and complete and clear patient instructions, including the importance of completing a course of ACT treatment and what to do if a dose is vomited or spat, should be given. The need to complete three sequential days of malaria treatment even if patients feel unwell or improve should be emphasised at both the community and clinic level.

## Consent

Written informed consent was obtained from the patient/parent/caretaker for the home questionnaires.

Verbal informed consent was obtained from the patient/parent/caretaker for the exit questionnaires.

## Supplementary material

Centre questionnaire

Home questionnaire

Exit questionnaire
