# Supplementary material for: Adherence to Artemisinin Combination Therapy for the treatment of uncomplicated malaria in the Democratic Republic of the Congo
Source: F1000Res. 2015 Apr 8;4:51. Originally published 2015 Feb 24. [Version 2] doi: 10.12688/f1000research.6122.2 (PMC4406189; doi:10.12688/f1000research.6122.2)
Supplement: Peer review history

# Peer review: Referee response for version 2

**DOI:** 10.5256/f1000research.6810.r8253

**Published:** 2015-04-13

**Authors:** Francois Nosten

**Affiliations:** 1 Nuffield Department of Clinical Medicine, University of Oxford, Oxford, UK

The authors should be congratulated for conducting this study in difficult circumstances. Given its very large experience, MSF must be encouraged to contribute more to the fight against malaria, with this type of operational research.

I have several comments and suggestions with regards to this study:

The authors wanted to investigate the reasons behind the rise in the number of malaria cases seen in their programs in DRC and set out to determine whether it may be caused by poor observance to the treatment.

In 2013 the clinic staff in Shamwana identified patients returning to the clinic with severe malaria after having been prescribed a course of ACT (artesunate-amodiaquine). It would be very useful to know more about these patients: where they from all age groups and both genders? What was the median time to the recurrent episode? How far from the clinic did they come from? What was the mean duration of fever before presentation? More generally it is important to have more details of the increasing number of cases see in the clinic between 2009 and 2013: age and gender distributions as well as the number of severe/uncomplicated cases.

There are several other potential reasons for the increase in cases: a larger population in the catchment area (or population movements), climatic changes, poor quality medicine, antimalarial resistance. Poor treatment observance is unlikely to be the only reason. The results of this study do not suggest that poor observance is a major problem, specially because the definition of probably non-adherence is too strict: taking the pills at a different time each day will not affect effectiveness. An interesting finding of the exit questionnaire is that few patients were given important additional information (Table 12). This suggests that the providers could probably improve the quality of the prescription. A useful indicator is the time taken by the prescribers to explain to the patients how to take the medicines.

Finding out the real contribution of poor observance is not too complicated. A dry blood spot (DBS) collected on all patients presenting with a positive RDT will be sufficient. One needs to know (from the clinic records) if and when the patient was treated last time. The DBS can be used to measure the concentration of amodiaquine in the blood as well as finding parasite DNA. Depending on the type of RDT used, there is a possibility of false positive in the days/weeks after a treatment with ACT because of the persisting HRP2 in the circulation. The DBS can be stored at room temperature and analyzed later. It may identify that in fact the problem of increasing cases is mostly due to the emergence of resistance to amodiaquine. In 2006 Swarthout and colleagues found a failure rate with ASAQ of 6.7% (5/74; 95% CI: 2.2-15.1) although in 2008-2009 another study (also from MSF) provided reassurance about the efficacy of ASAQ (cure rate 98.3 (94.1-99.8) by day 42.

One suggestion to improve the impact of the intervention (LLIN and ACT) is to ensure that the diagnostic (RDT) and treatment (ACT) is given as soon as possible after the beginning of fever (<48h). This is because in order to prevent transmission, patients must be treated before the emergence of mature gametocytes in the circulation. The addition of the low single dose primaquine (as recommended by WHO) will help reducing transmission.

In conclusion, this is a useful paper, but some more epidemiological details about the case-load and some simple investigations would help understanding the factors behind the increasing number of malaria cases seen in MSF clinics. Setting up malaria posts in the villages, where people can go early in the course of their fevers would help maximizing the impact on transmission.

I have read this submission. I believe that I have an appropriate level of expertise to confirm that it is of an acceptable scientific standard.

# Peer review: Referee response for version 1

**DOI:** 10.5256/f1000research.6558.r7807

**Published:** 2015-03-02

**Authors:** Anne Moore

**Affiliations:** 1 Department of Pharmacology and Therapeutics, University College Cork, Cork, Ireland

This is an interesting paper from MSF epidemiologists that aims to determine some of the reasons for significant increases in malaria cases in DRC. It is imperative to prevent the emergence of anti-malarial drug resistance and patient adherence and compliance is critical to this issue. The data presented here provides evidence of what is happening in the field and touches on patients’ attitudes and knowledge in this area. The authors focus on patient compliance and statistically examine socio-demographic and other factors for non-adherence to the prescribed time schedule and dosage. A key finding is the low rate of adherence, which has significant impact on drug-based treatment of malaria. Non-adherence due to vomiting or feeling unwell in a proportion of individuals is an important finding for the drug formulation community; how can this be overcome by changing the formulation or possibly the route,  in the future?  Timing was also highlighted as an obstacle to correct drug administration; again this is an obstacle that should be tackled both by drug developers, practitioners in the field and those interested in health literacy. The authors should consider minor revisions to their paper to discuss these issues in more depth. For example, based on their data, what are the next critical steps that should be focused on; from both a practice, and more long-term, drug design perspective? How can we succeed in patient education relating to the importance of adherence and designing and implementing a system that results in better patient adherence? Given that the authors found that gender is a significant factor; can the authors suggest how this finding should be used to develop strategies to improve patient compliance? Finally, the authors discuss the problems of trying to compare across studies due to different study designs and methods. From a methodology perspective, therefore, it would be useful if the authors included the full questionnaires used in this study as an appendix, with the objective of replication of the study in other areas.

I have read this submission. I believe that I have an appropriate level of expertise to confirm that it is of an acceptable scientific standard.
